# Curdlan β-1,3-Glucooligosaccharides Induce the Defense Responses against *Phytophthora infestans* Infection of Potato (*Solanum tuberosum* L. cv. McCain G1) Leaf Cells

**DOI:** 10.1371/journal.pone.0097197

**Published:** 2014-05-09

**Authors:** Jing Li, Li Zhu, Guangxing Lu, Xiao-Bei Zhan, Chi-Chung Lin, Zhi-Yong Zheng

**Affiliations:** 1 Key Laboratory of Carbohydrate Chemistry and Biotechnology of Ministry of Education, Jiangnan University, Wuxi, Jiangsu, China; 2 Jiangsu Rayguang Biotech Company, Ltd., Wuxi, Jiangsu, China; University of Texas, United States of America

## Abstract

Activation of the innate immune system before the invasion of pathogens is a promising way to improve the resistance of plant against infection while reducing the use of agricultural chemicals. Although several elicitors were used to induce the resistance of potato plant to microbial pathogen infection, the role of curdlan oligosaccharide (CurdO) has not been established. In the current study, the defense responses were investigated at biochemical and proteomic levels to elucidate the elicitation effect of CurdOs in foliar tissues of potato (*Solanum tuberosum* L. cv. McCain G1). The results indicate that the CurdOs exhibit activation effect on the early- and late-defense responses in potato leaves. In addition, glucopentaose was proved to be the shortest active curdlan molecule based on the accumulation of H_2_O_2_ and salicylic acid and the activities of phenylalanine amino-lyase, β-1,3-glucanase and chitinase. The 2D-PAGE analysis reveals that CurdOs activate the integrated response reactions in potato cells, as a number of proteins with various functions are up-regulated including disease/defense, metabolism, transcription, and cell structure. The pathogenesis assay shows that the ratio of lesion area of potato leaf decreased from 15.82%±5.44% to 7.79%±3.03% when the plants were treated with CurdOs 1 day before the infection of *Phytophthora infestans*. Furthermore, the results on potato yield and induction reactions indicate that the defense responses induced by CurdOs lasted for short period of time but disappeared gradually.

## Introduction

As one of the most important economic plant, potato (*Solanum tuberosum* L.) has been widely cultivated around the world for centuries. The potato yield is frequently affected by devastating diseases caused by pathogens such as *Phytophthora infestans* [1], *Erwinia carotovora* [2], and *Verticillium dahliae* [3]. Historically, the reduction of potato yield caused by *P. infestans* led to the tragic Great Famine in the 1840s in Ireland [4]. Bactericide and fungicide overload the environmental system despite the alleviation of potato diseases. It is an important issue to protect potato plants against infection of pathogens using eco-friendly methods instead of applying various agricultural chemicals. An effective method is to activate the plant innate immune defense system prior to the infection of pathogens [5]. The defense-related phytoalexins [6] and/or pathogenesis-related proteins (PRs) [7] in the plant tissues possess exquisite defense activities responding to pathogen invasion [8].

β-1,3-Glucans are recognized as elicitors for plant defense responses [9]. Curdlan, a linear water-insoluble β-1,3-glucan produced by fermentation of *Agrobacterium* sp., is approved as a safe food additive [10]. Recently, we have developed an effective process to produce curdlan β-1,3-oligosaccharides [11] which exhibit various biomedical functions [12]. Although the mixture of curdlan oligosaccharides (CurdOs) activates the defense response in tobacco (*Nicotiana tabacum* L.) cells [13], the elicitation effect of specific oligosaccharide on potato (*S. tuberosum* L.) tissues and the shortest active CurdO molecule have not been determined until now.

The plant cell perceives pathogens and responds in a cascade pattern. The ion fluxes and accumulation of reactive oxygen species are triggered within minutes, and then a series of reactions are activated, such as the stimulation of phenylpropanoid pathway, generation of signal molecules (salicylic acid or jasmonates, etc.), and accumulation of PRs and/or phytoalexins [5], [14]–[17]. Phenylpropanoid compounds released from the phenylpropanoid pathway perform physical and chemical barriers against the invasion of pathogens and also activate the local and systemic signaling for the induction of responding genes. As the crucial enzyme, phenylalanine ammonia-lyase (PAL) catalyzes the transformation from L-phenylalanine to *trans*-cinnamic acid [16], [18]. Both β-1,3-glucanase (GLU) and chitinase (CTN) are important PRs in the plant immune system [7], [19]–[20]. Consequently, detailed elucidation of the elicitor activities of CurdOs in the potato foliar tissues is necessary. Several factors are detected in this study, including the accumulation of hydrogen peroxide (H_2_O_2_) and salicylic acid (SA). The enzymatic activities of PAL, GLU, and CTN are also determined.

The two-dimensional polyacrylamide gel electrophoresis (2D-PAGE) is an appropriate method for the analysis of protein expression profiles in plant cells. 2D-PAGE has been previously applied on the potato-related projects which include the analysis of potato tuber bruising effect [21], investigation of the fertilization regimes [22], and the proteome profiling of potato leaves after wounding [23]. In this study, the 2D-PAGE method was performed to compare the expression profiles of the proteins in potato leaf cells after treatment with various elicitors.

In view of its non-polluting nature to the environment, it is far better to establish the elicitation properties of curdlan oligosaccharides to develop eco-friendly antifungal natural products for plant protection. The objective of the current study is to verify the elicitation ability of CurdOs in potato (*S. tuberosum* L. cv. McCain G1) foliar tissues at the biological and proteomic levels. Moreover, experiments were also designed to identify the shortest curdlan molecule capable of triggering defense response in potato cells. In addition, the pathogenesis assays against *P. infestans* infection were also performed.

## Materials and Methods

### Materials and Chemicals

Laminarin (from *Laminaria digitata*), Chitin azure, *trans*-cinnamic acid, and salicylic acid were purchased from Sigma (St. Louis, MO, USA). Curdlan was purchased from Takeda Chemical Industries, Ltd. (Osaka, Japan). Other materials and chemicals were all analytical grade, and purchased from Sinopharm Chemical Reagents Co. Ltd. (Shanghai, China).

### Plant, pathogen treatment and growth conditions

All potato (*S. tuberosum* L. cv. McCain G1) plants were grown in a greenhouse under controlled conditions (photoperiod of 16 h every day, 24°C). Eight-week-old plants were used for experiments. *P. infestans* strain JN (weakly aggressive) was purchased from Guangdong Culture Collection Center (Guangzhou, China). The cultivation and zoospores collection methods were described previously [24].

### Preparation of curdlan β-1,3-oligosaccharides (CurdOs)

The CurdOs were prepared as described by us previously [11]. Briefly, 10 g/L of curdlan-water suspension was hydrolyzed by 1 M sulfuric acid (H_2_SO_4_). Subsequently, barium hydroxide (Ba(OH)_2_) was added to neutralize the solution to pH 7. The hydrolyzate was filtered with a 0.22 µm nylon membrane, and then fractionated with a Hitachi CM5000 HPLC system (Tokyo, Japan) equipped with a Click Maltose column (7.8 mm×150 mm) and a refractive index detector (RID). The mobile phase was acetonitrile-water (7∶3, v/v) at 1.50 mL/min flow rate. Purified CurdOs with various degrees of polymerization (DP) were separately collected and dried with the vacuum centrifugal concentrator (CentrlVap, Labconco Corp., Kansas City, MO, USA). CurdOs were stored at 4°C for later use.

### Treatment of potato seedlings with elicitors

CurdOs (DP 3, glucotriose, DP 4 glucotetraose, DP 5, glucopentaose, DP 6, glucohexaose, and DP 7, glucoseptaose) and laminarin (Lam) were used as elicitors in the experiments.

Potato leaves were cleaned by spraying sterilized water 2 hours before the infiltration of elicitors. Subsequently, 10 µg of elicitor (40 µL, 250 µg/mL) was gently coated on the surface of every potato leaf. In addition, the leaves treated with 40 µL purified water without elicitors were used as control (CK). Three potato seedlings cultivated in one pot were used for the analysis of each elicitor. The entire leaf was processed for the analyses of H_2_O_2_ and SA accumulation and assessment of enzymatic activities. All tests were performed in triplicates. A total of 201 leaves were used in the experiment.

### Pathogenesis assay with P. infestans and potato yield evaluation

The mixture of CurdOs from DP 3 to DP 7 was used as elicitor. Potato plants were cultivated as aforementioned and were treated in three different manners including 1) plants sprayed with 10^5^ mL^−1^ of *P. infestans* zoospores at 45-d of cultivation (*P. infestans*), 2) plants treated with 250 µg/mL CurdOs 1 day before *P. infestans* treatment (1 d+*P.i.*), and 3) plants treated with 250 µg/mL CurdOs 5 days before *P. infestans* treatment (5 d+*P.i.*). *P. infestans* infection was characterized as the leaf lesion area in the experiment. A total of 30 potato leaves were randomly selected in each treatment two weeks after the spray of the pathogen. The whole leaf area and lesion area were measured with Area tool in Adobe Acrobat (Version 7.0.9, Adobe Systems Inc., USA). The ratio of lesion area (RLA) was calculated as follows:

RLA = (Lesion area)/(Whole leaf area)×100%

Potatoes were harvested after 3-month of cultivation to determine the whole plant yield and average potato weight. A total of 60 potato plants were used for the analysis.

### Statistical analysis

Statistical data analyses were performed with SPSS version 18.0 (SPSS Inc., Chicago, IL, USA). One-way ANOVA was used to compare the response factors (accumulation of H_2_O_2_ and SA, and enzymatic activities of PAL, GLU, and CTN) on elicitor treated and control potato plants. One-way ANOVA was also used to compare the ratio of leaf lesion area, whole plant yield, and average potato weight of pathogen infected plants with the elicitor treated plants. Means were compared by the least significant difference (LSD) tests at *p*<0.05.

### Determination of hydrogen peroxide (H_2_O_2_) level in potato leaves

The accumulation of H_2_O_2_ in the leaves was determined as described previously [25] with slight modification. Briefly, freshly cut potato leaves (200 mg) were ground in liquid nitrogen to fine powder and blended with 1.0 mL pre-cold acetone for 5 min. The mixture was centrifuged for 10 min at 8,000 *g* to remove the pellet. Then 0.8 mL of supernatant was added to 0.1 mL titanium reagent (20.2% HCl containing 20% TiCl_4_) and 0.2 mL (25–28%) concentrated ammonia water. The mixture was centrifuged for 10 min at 3,000 *g* to remove the supernatant. The pellet was washed 3 times with acetone to remove the pigments. The pellet was then dissolved into 2.0 mL H_2_SO_4_ (2 M). The final solution was used for measuring relative absorbance at 415 nm for determination of the concentration of H_2_O_2_ in triplicates.

### Determination of the level of salicylic acid (SA)

The method for the analysis of SA was adopted as described by van Spronsen *et al.* [26] with slight modification. Briefly, the quantification of SA was performed with a Hitachi CM5000 HPLC system which equipped an Agilent Zorbax SB-C18 column (4.6 mm×300 mm). The optimal eluent was 50 mM sodium acetate, pH 5.5, blended with methanol (85∶15, v∶v). The flow rate was 1.00 mL/min. A spectrofluorometric detector was used with an excitation wavelength of 305 nm and emission wavelength of 410 nm. All tests were performed in triplicates.

### Assays of pathogenesis-related proteins (PRs) activities

Potato leaves (200 mg fresh weight (FW)) were ground in liquid nitrogen to fine powder and added into 1 mL protein extraction solution (50 mM sodium acetate, pH 5.5, 5 mM EDTA, 2 mM DTT, 0.04% sodium thiosulfate, 0.1% polyvinylpyrrolidone) and blended at 4°C for 10 min. The resulting mixture was centrifuged at 10,000 *g* for 30 min at 4°C to remove the pellet. The supernatant was the crude protein extract which was stored at −20°C for later use.

For the determination of β-1,3-glucanase (GLU) activity, the reaction system was composed of 200 µL of 10 g/L laminarin (in 50 mM sodium acetate, pH 5.0), 200 µL of 50 mM sodium acetate buffer (pH 5.0), 100 µL of protein extract. The reaction solution was incubated at 37°C for 2 h. The hydrolyzate was then heated to 100°C for 10 min to inactivate the enzyme. One GLU activity unit (1 U) was defined as the quantity of protein that hydrolyzes the substrate to release 1 µg of reducing sugar per minute.

For the determination of chitinase (CTN) activity, the reaction system was composed of 140 µL of 25 g/L chitin azure (in 50 mM sodium acetate, pH 5.0), 70 µL of protein extract. The reaction solution was incubated at 37°C for 2 h. Then 100 µL of 2 M HCl was added into the system to stop the reaction. Assessment of chitinase hydrolyzate was made using the method of Ippolito *et al.* [27].

For the determination of phenylalanine ammonia-lyase (PAL) activity, the previously reported method [28] was applied with slight modification. Briefly, the reaction system was composed of 500 µL of 20 mM L-phenylalanine (in 50 mM Tris-HCl, pH 8.5), 400 µL of 50 mM Tris-HCl buffer (pH 8.5), 100 µL of protein extract. The reaction solution was incubated at 37°C for 2 h. Then 200 µL of 6 M HCl was added into the system to stop the reaction. The product *trans*-cinnamic acid was extracted by 2 mL diethyl ether twice. After evaporation of the solvent, the solid residue was resuspended in 500 µL methanol. The product was quantified by spectrometry at 290 nm.

All experiments were performed in triplicates.

### Two-dimensional polyacrylamide gel electrophoresis (2D-PAGE)

Leaf protein extract was incubated with 10% TCA in acetone overnight at −20°C. The precipitated proteins were centrifuged at 10,000 *g* for 20 min at 4°C to recover the pellet, and washed with ice-cold acetone 3 times. The pellet was resuspended in the sample rehydration solution (8 M urea, 2% CHAPS, 1% IPG buffer, 20 mM DTT, 0.002% bromophenol blue). The undissolved substances were removed by centrifugation at 10,000 *g* for 20 min.

Isoelectric focusing (IEF) was carried out with 18 cm IPG strips (pH 3–10, linear) in an IPGPhor system (GE healthcare, Piscataway, NJ, USA). The IPG strip was rehydrated with 350 µL rehydration solution containing 200 µg proteins for 12 h. A constant voltage protocol was applied for all the experiments, including 100 V for 2 h, 250 V for 2 h, 500 V for 1 h, 1,000 V for 1 h, 4,000 V for 2 h, 8,000 V for 2 h, and finally 8,000 V to a total 80,000 Vh. After IEF, the IPG strips were denatured with equilibration buffer (50 mM Tris-HCl, pH 8.8, 6 M urea, 30% glycerol, 2% SDS, 0.002% bromophenol blue) containing 1% DTT for 15 min at room temperature and, consecutively, incubated with the equilibration buffer containing 2.5% iodoacetamide for 15 min. The second dimension was performed on a 12.5% polyacrylamide gel using an Ettan DALTsix system (GE healthcare, Piscataway, NJ, USA). After electrophoresis, the gels were stained with Coomassie Brilliant Blue G250 solution at room temperature overnight, and then destained with deionized water.

### Spots selection and in-gel digestion

The gels were scanned using a Personal Densitometer system (GE healthcare, Piscataway, NJ, USA). The gel images were processed using the BioRad PDQuest 2-D analysis software (Ver. 8.0.1, Bio-Rad Laboratories, Inc., Hercules, CA, USA). The protein content was calculated based on the color intensity on the gels from two replicates.

The target protein spots were excised for the in-gel digestion. The digestion method was adopted as described by Xu *et al.* [29]. After digestion by trypsin at 37°C overnight, the resulting tryptic fragments were collected and vacuum dried for the MALDI-TOF/TOF analysis.

### MALDI-TOF/TOF mass spectrometry and protein identification

The method for mass spectrometry analysis was described previously [21]. 0.7 µL of protein fragments and 0.7 µL of HCCA (matrix) were spotted on a MTP AnchorChip 384 T F (Bruker Daltonics Inc., Bremen, Germany), respectively. After drying, the samples were processed and the peptide mass fingerprint data were collected by an Ultraflextreme MS spectrometer (Bruker Daltonics Inc.). The LIFT fragmentation method was used to obtain peptide MS/MS spectra. Peptide sequences were analyzed automatically by the PepSeq software. The Mascot search engine was used to identify the protein (http://www.matrixscience.com) by searching from the NCBI non-redundant database (http://www.ncbi.nlm.nih.gov). The criteria for positive identification were followed the method by Xu *et al*. [29].

## Results

### Curdlan oligosaccharides elicit defense responses in potato leaf cells

**Figure 1:**
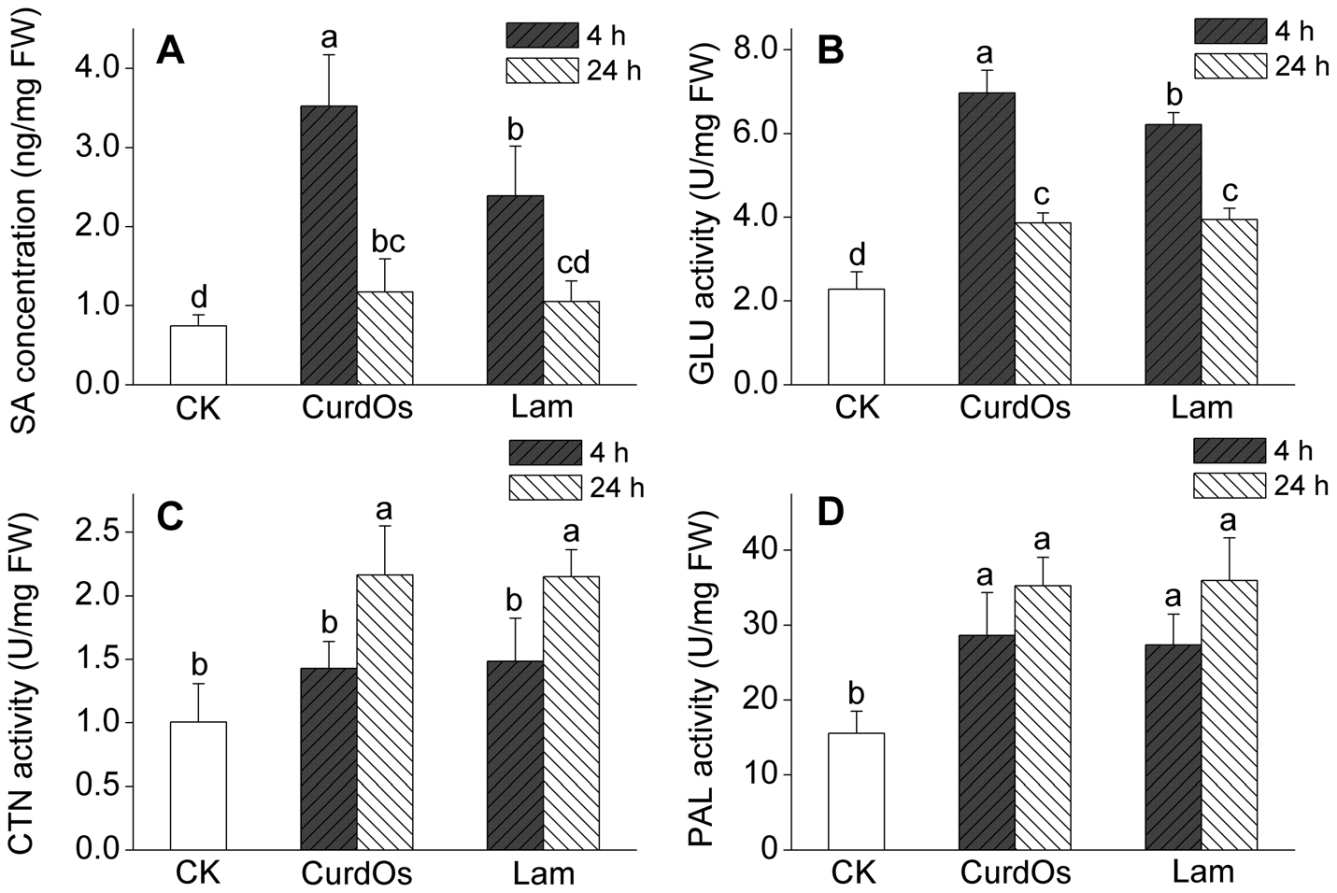
Defense reactions of potato (*S. tuberosum* L. cv. McCain G1) in response to elicitors. The potato leaves were treated separately with curdlan oligosaccharides (CurdOs, a mixture of glucotriose, glucotetraose, glucopentaose, glucohexaose, and glucoseptaose) and laminnarin (Lam). Sampling was done at 4 h and 24 h after elicitation for each set. Potato leaves treated with deionized water were used as control, CK. All tests were performed in triplicates. SA, salicylic acid; GLU, β-1,3-glucanase; CTN, chitinase; PAL, phenylalanine ammonia-lyase; FW, fresh weight. Values are means±SD. Lowercase letters indicate significant differences (LSD test at *p*<0.05).

To elucidate the elicitation effect, the mixture of CurdOs (from DP 3 to DP 7) was applied to the potato leaves. The defense responses *in vivo* were detected at 4 h and 24 h after elicitation (Fig. 1). After treatment with CurdOs, the concentration of signal molecule salicylic acid (SA) increased substantially 4 h after elicitation, which was higher than that treated with laminarin (Lam). Both the elicitors positively activated the accumulation of SA significantly comparing to the blank control (Fig. 1A, CK), whereas at 24 h the accumulation of SA remarkably decreased. Although the SA level detected in Lam set was higher than that in CK set, the difference was not significance. However, the SA level in CurdOs set after 24 h elicitation still showed significant increment than CK set. The level of enzymatic activity of GLU significantly improved at 4 h after CurdOs or Lam treatment, which was similar to the SA results. In addition, the GLU level at 24 h after elicitation remained higher than the CK set.

Both CurdOs and Lam improved the activities of CTN and PAL in leaf cells especially at 24 h after treatments. When the leaves were treated with CurdOs, the activities of PAL and CTN elevated up to 126% and 115%, respectively, comparing to the elicitor-free control at 24 h (Fig. 1, C and D).

As stated above, SA, GLU, CTN, and PAL are all plant defense response-related molecules, the results show that the curdlan oligosaccharides mixture exhibited elicitation effect on inducing the defense responses in potato leaf cells.

### Glucopentaose is the shortest active curdlan oligosaccharide

**Figure 2:**
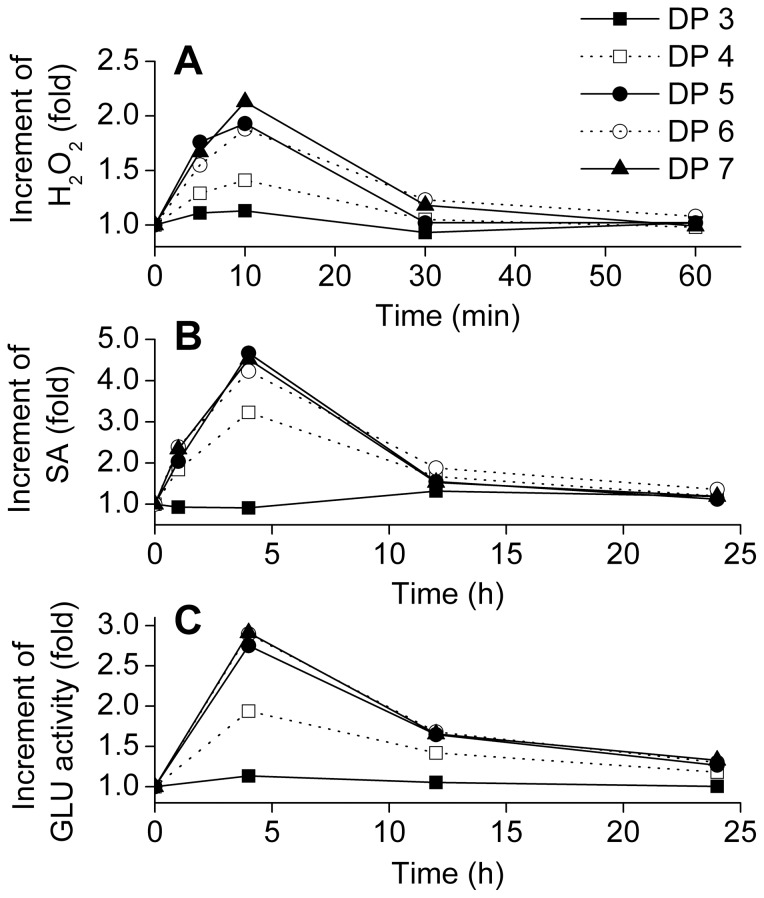
Time-course increment of H_2_O_2_, salicylic acid (SA), and β-1,3-glucanase (GLU) in potato leaves treated with curdlan oligosacchrides. The concentration of H_2_O_2_ and SA and the activity of GLU were set as 1.0 before elicitation (0 min). The values after elicitation were expressed as the increment of fold(s) comparing with the values before elicitation. The curdlan oligosaccharides used in this experiment have the degree of polymerization (DP) from 3 to DP 7. All the tests were performed in triplicates.

**Figure 3:**
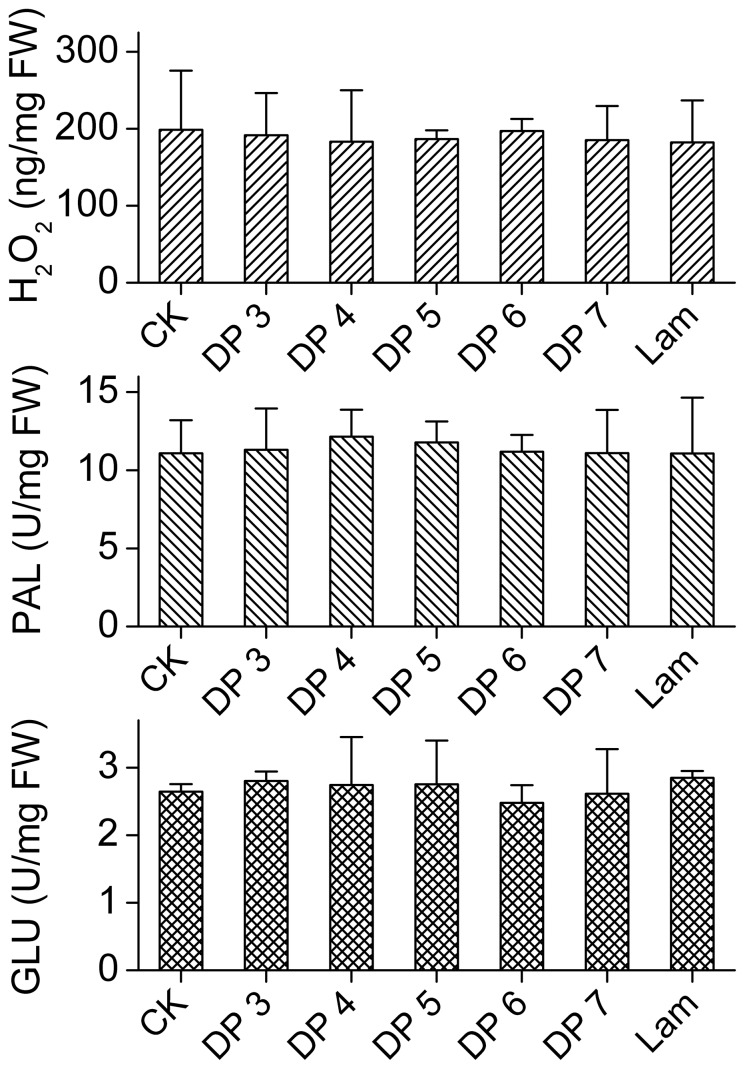
Long-term defense activities of potato in response to elicitors. The potato leaves were treated with various curdlan oligosaccharides (degree of polymerization (DP) from 3 to DP 7). Sampling was done at 20 d after elicitation for each set. Potato leaves treated with deionized water were used as elicitor-free control, CK. All tests were carried out in triplicates. PAL, phenylalanine ammonia-lyase; GLU, β-1,3-glucanase. Values are means±SD. Lowercase letters indicate significant differences (LSD test at *p*<0.05).

To determine the changes of the defense factors, potato plants were separately treated with various curdlan oligosaccharides (from DP 3 to DP 7). Accumulations of H_2_O_2_ and SA and the enzymatic activity of GLU were detected in potato leaves (Fig. 2). Within minutes, the early-defense response was observed, as the concentration of H_2_O_2_ increased considerably 5 min after elicitation and reached the maximum level at 10 min. The peak values of SA and GLU were both observed at 4 h. The response time for SA and GLU was much longer than H_2_O_2_, which indicated that the late-defense response was occurring in the cells. (Fig. 2, B and C). Additionally, the levels of all the three factors dropped gradually when the elicitation time prolonged which indicated that the CurdOs effect only lasted a short time in the leaf cells. Therefore, the H_2_O_2_ concentration, GLU and PAL activities were determined at 20 d after elicitation (Fig. 3). No significant difference was observed between the CurdOs treated plants and blank control (*F*_(6,14), H2O2_ = 0.050, *p*_H2O2_ = 0.999; *F*_(6,14), GLU_ = 0.230, *p*_GLU_ = 0.960; *F*_(6,14), PAL_ = 0.098, *p*_PAL_ = 0.995), which confirmed the conclusion that CurdOs activated the short-term elicitation effect in potato leaf cells.

**Figure 4:**
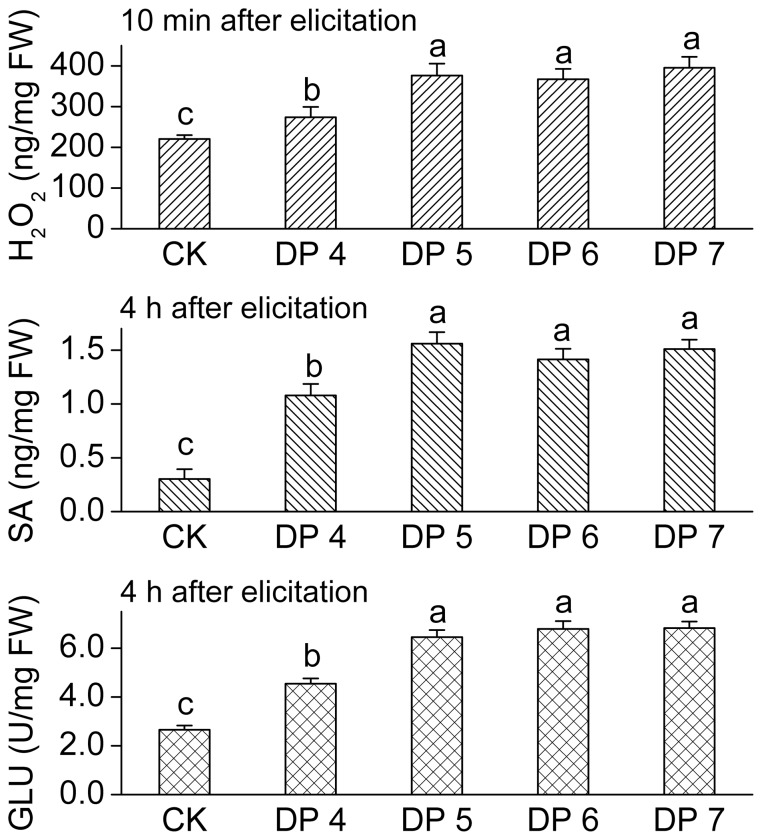
Induction of H_2_O_2_, salicylic acid (SA) and β-1,3-glucanase (GLU) in potato leaves. Potato plants were separately treated with various curdlan oligosaccharides (degree of polymerization (DP) from 4 to DP 7). The accumulation of H_2_O_2_ was detected after elicitation at 10 min. Both the SA concentration and GLU activity were detected at 4 h. CK, elicitor-free control. Values are means±SD. Lowercase letters indicate significant differences (LSD test at *p*<0.05).

Although the CurdOs induced the responses in potato cells, the induction strength (expressed as the increment of fold) varied in accordance with the different DPs (Fig. 4). DP 3 had only slight effect on the elicitation, whereas the other oligosaccharides showed remarkable effect. As shown in Figure 4, DP 5, DP 6, and DP 7 have the similar induction capability (*p*_H2O2_ = 0.148, *p*_SA_ = 0.255, *p*_GLU_ = 0.295) which is significantly higher than DP 4 and the control sets (*F*_(4,10), H2O2_ = 32.483, *p*_H2O2_<0.01; *F*_(4,10), SA_ = 83.757, *p*_SA_<0.01; *F*_(4,10), GLU_ = 150.140, *p*_GLU_<0.01). Accordingly, it is concluded that β-1,3-glucopentaose (DP 5) is the essential linear molecule to fully elicit the defense responses in potato leaves.

### CurdOs induce the whole cell responses in potato foliar tissues

**Figure 5:**
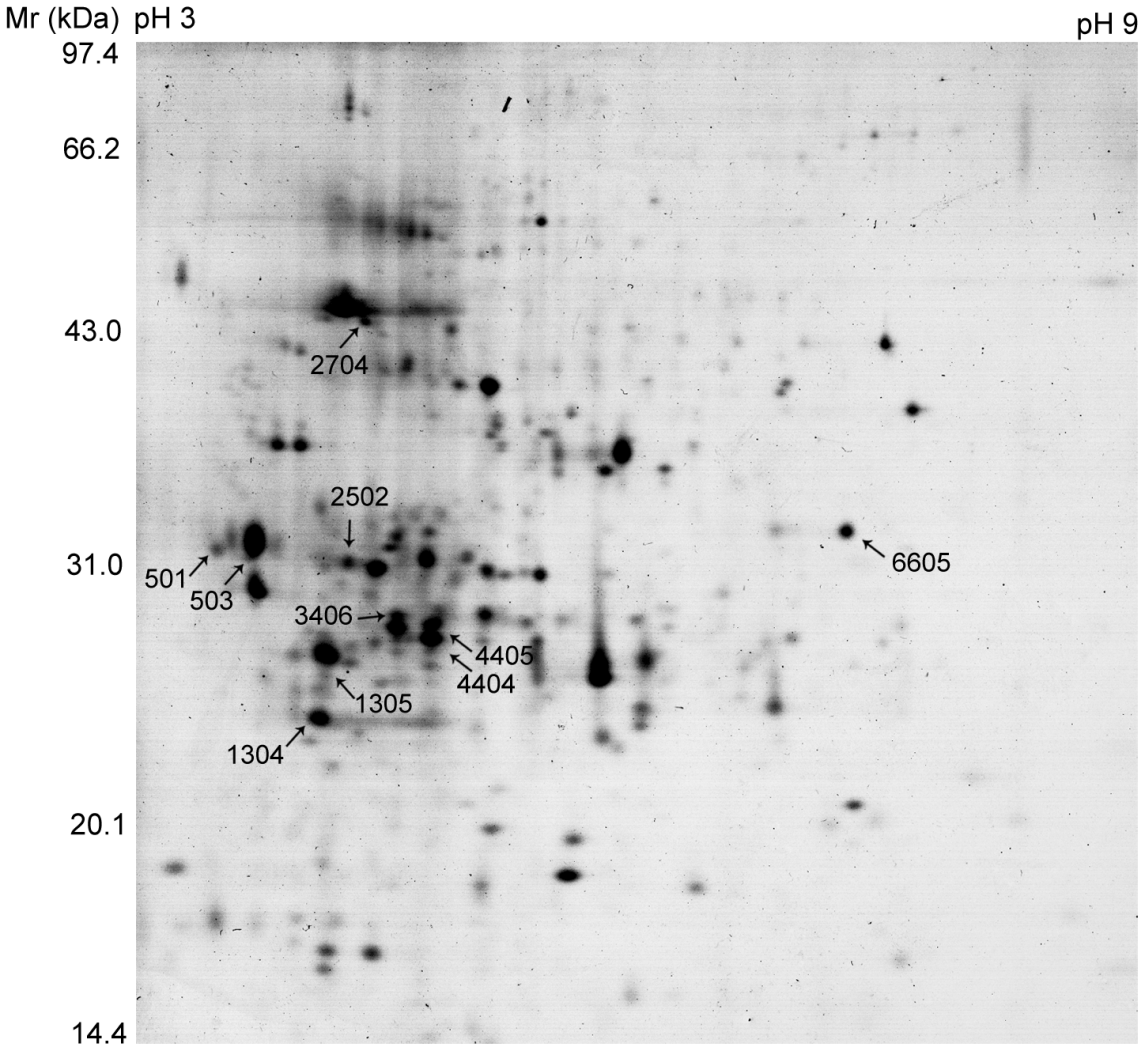
Coomassie Brilliant Blue stained 2D-PAGE gel of proteins in potato leaf cells treated with glucopentaose. 2D-PAGE separation was performed in the first dimension on a linear pH 3–10 IPG strip, and in the second dimension on a 12.5% polyacrylamide gel. The spots were numbered automatically by the Bio-Rad PDQuest software. The gel ranging from pH 9 to pH 10 was truncated as no visually detected spot was found within this region.

2D-PAGE experiments were performed to analyze the changes of protein expression. Potato leaves were sampled at 12 h after elicitation and further separated by 2D-PAGE. After staining, more than 150 spots displayed on each gel (Proteins extracted from the glucopentaose-treated plant (DP 5) was shown as an example, Fig. 5.). Ten up-regulated proteins were further analyzed based on the color intensity.

**Figure 6:**
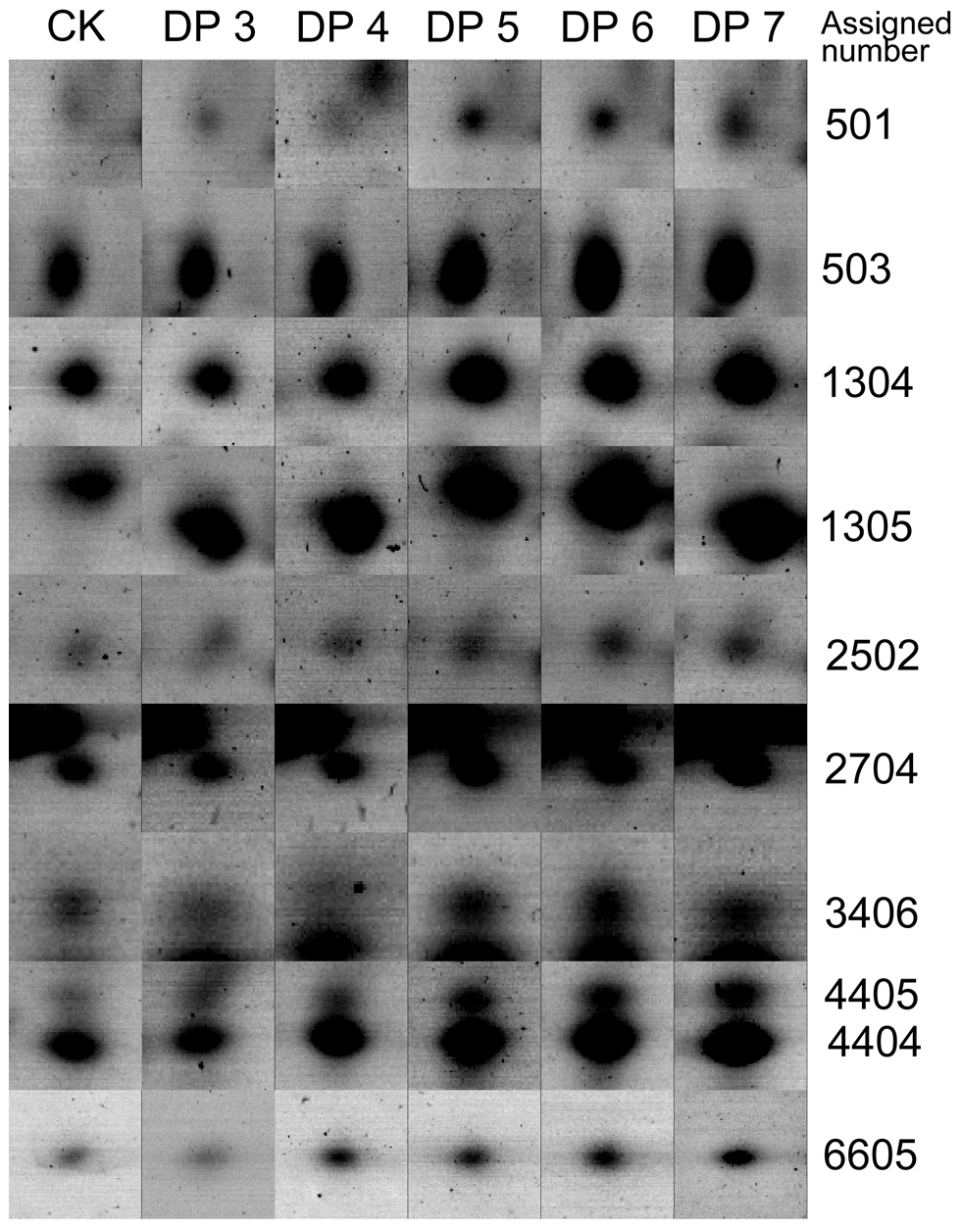
Comparison of protein spots from the elicitor-treated sets with the elicitor-free control. Proteins were extracted from the leaf cells at 12-free sample (blank control). DP, degree of polymerization.

As shown in Figure 6, the spot intensity increased along with the increment of DP of the curdlan oligosaccharide. In addition, the spot intensity kept visually stable from DP 5 to DP 7. A quantitative analysis of the spots intensity (illustrated as Figure S1) confirmed the observation that glucopentaose (DP 5) was the shortest active elicitor for potato plant treatment.

Furthermore, these 10 proteins were identified by the MALDI-TOF/TOF methodology. The functional category of protein was classified in accordance with the method described previously [30] (Table 1). The identified proteins involved in several functions, including metabolism, transcription, cell structure and disease/defense. The results indicate that the CurdOs trigger the complex responses in potato leaf cells.

**Table 1:** Proteins identified from the potato (*S. tuberosum* L. cv. McCain G1) leaf cells by MALDI-TOF/TOF mass spectrometry.

AN a	Protein identification	Species	Accession No. b	MOWSE score c	Functional category
501	Predicted: neurochondrin-like	*Solanum lycopersicum*	gi|460398801	75	Unclear classification
503	At1g69830/T17F3_14-like	*Solanum tuberosum*	gi|53689726	94	Metabolism
1304	Thioredoxin peroxidase	*Nicotiana tabacum*	gi|21912927	334	Secondary metabolism
1305	Putative kinesin heavy chain	*Arabidopsis thaliana*	gi|4314358	81	Cell structure
2502	Leucine aminopeptidase, partial	*Solanum tuberosum*	gi|21487	83	Metabolism
2704	Acetylornithine deacetylase	*Arabidopsis thaliana*	gi|42566909	146	Metabolism
3406	Homeobox protein PpHB7	*Physcomitrella patens*	gi|21623495	90	Transcription
4404	Putative Kunitz-type invertase inhibitor	*Solanum tuberosum*	gi|327198777	70	Disease/defense
4405	Protein z-type serpin	*Hordeum vulgare* subsp. *vulgare*	gi|1310677	193	Disease/defense
6605	Endochitinase	*Solanum tuberosum*	gi|1705805	180	Disease/defense

The functional category was classified based on the published method [30].

a , AN: assigned number for the protein spot;

b , Accession No.: Accession number from NCBI (http://www.ncbi.nlm.nih.gov);

c , MOWSE score: MOlecular Weight SEarch score, The score >64 indicates identity or extensive homology (*p*<0.05).

### CurdOs decrease the ratio of lesion area on potato leaves caused by P. infestans

It is proven that the CurdOs are capable of inducing the early- and late-defense responses in potato leaf cells. However, it is still uncertain that whether the CurdOs protect the potato plants from pathogen infection under cultivation conditions.

**Figure 7:**
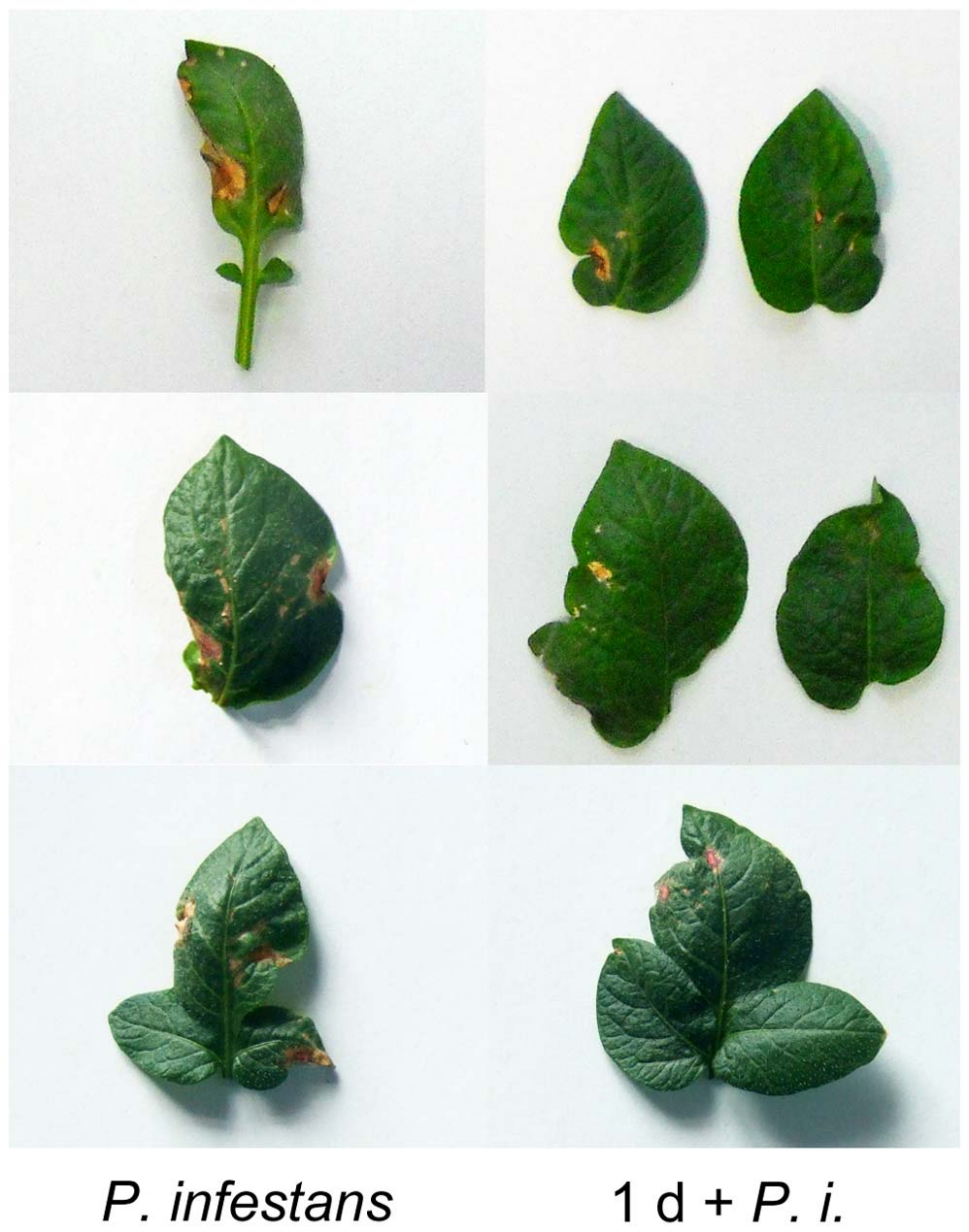
Lesion area of the potato leaf infected with *P. infestans* pathogen and curdlan oligosaccharides treatments. *P. infestans*, plants were treated with 10^5^ mL^−1^ of *Phytophthora infestans* zoospores at 45-day of cultivation. 1 d+*P.i.*, 250 µg/mL curdlan oligosaccharides (CurdOs, the mixture of glucopentaose, glucohexaose, and glucoseptaose) was sprayed on the potato leaves at 1 d before *P. infestans* treatment. Potato leaves were cut off from the plants and imaged with digital camera.

*P. infestans* zoospores suspension (10^5^ mL^−1^) was sprayed on the potato leaves at 45 d of cultivation. The mixture of CurdOs (glucopentaose, glucohexaose, and glucoseptaose) was infiltrated into the potato leaves prior to the infection of *P. infestans*. Figure 7 shows the comparison of lesion area of CurdOs-free leaves (left) with CurdOs-treated leaves (right). The ratio of lesion area (RLA) decreased when the plants were treated with CurdOs at 1 d before infection of the pathogen. *P. infestans* caused an average of 15.82%±5.44% RLA on leaves, whereas the RLA dropped to 7.79%±3.03% when leaves were treated with CurdOs indicating that the mixture of CurdOs triggered an effective defense response in potato plants and protected the plants from *P. infestans* infection (*t′* = 6.191, *t*_0.01(45)_ = 2.690, *p*<0.01). In addition, the leaves which were treated with CurdOs didn't show visible difference with the control without treatment (data not shown).

**Figure 8:**
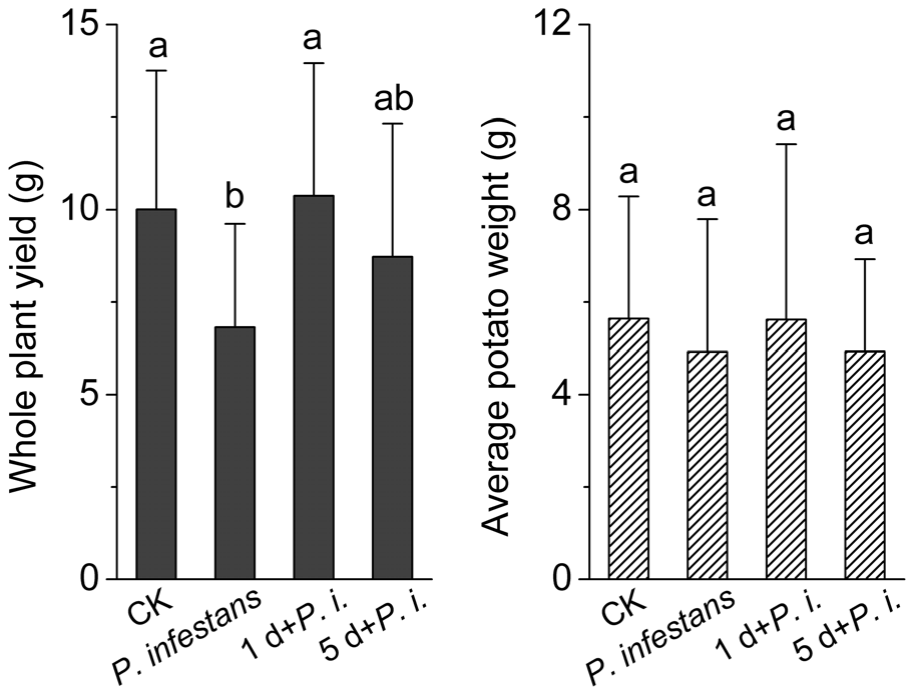
Potato yield under various treatments. Potatoes were harvested and weighed after 3-month cultivation. *P. infestans* infected plants were treated by 10^5^ mL^−1^ of *Phytophthora infestans* zoospores at 45-day of cultivation. 1 d+*P.i.*, 250 µg/mL curdlan oligosaccharides (CurdOs, the mixture of glucopentaose, glucohexaose, and glucoseptaose) was sprayed on the potato leaves 1 d before *P. infestans* treatment. 5 d+*Pi*, 250 µg/mL CurdOs was used 5 d before *P. infestans* treatment. CK, control without treatment. Values are means±SD. Lowercase letters indicate significant differences (LSD test at *p*<0.05).

The potato yield after 3-month of cultivation was determined (Fig. 8). When treated with *P. infestans*, the whole plant yield of potato after harvest reached 6.82±2.80 g. Significant increase of potato yield was observed when the CurdOs were applied 1 d prior to *P. infestans* treatment (10.37±3.75 g, *t′* = 2.741, *t*_0.05(22)_ = 2.074, *p* = 0.012). However, the potato yield did not show significant difference from the *P. infestans* set when the plants were treated with CurdOs 5 d before the infection of the pathogen (*p* = 0.146). In comparison with the control set (CK), the potato plant yield in 1 d+*P. i.* set kept at the same level, which indicates that the CurdOs treatment shows protection effect on the plants. In addition, the average potato weight was not affected by the application of CurdOs. The results support the conclusion that CurdOs activate the potato defense responses against *P. infestans* infection for a short period of time.

## Discussion

### Curdlan oligosaccharides induce a series of early- and late-defense responses in potato leaf cells

It is reported that the hepta-β-glucan has the ability to elicit the synthesis of phytoalexin in soybean [31]. The hepta-β-glucan consisting of the five-connected β-1,6-glucosyl backbone and two β-1,3-glucosyl branches is the minimal structure required for the induced response in soybean [32]. Whereas Klarzynski *et al*. [33] has shown the shortest β-glucan with elicitor activity in tobacco cells is laminaripentaose. Therefore, the recognition patterns for elicitors might vary in different plants. Although several studies were conducted to elucidate the defense responses in potato tissues, the elicitors were focused on the branched glucans or oligosaccharides, such as, α-glucan [34]–[35], *Phytophthora infestans* glucans [24], [36]. Hitherto, the evaluation of curdlan oligosaccharides on potato defense response has not been reported.

A variety of proteins have been identified as pathogenesis-related proteins (PRs) and classified based on their functions into 14 families [7], [17], [20]. β-1,3-Glucanase belongs to the PR-2 Family, whereas chitinases are in the PR-3, -4, -8, or -11 Families. Phenylalanine ammonia-lyase which catalyzes the transformation from L-phenylalanine to *trans*-cinnamic acid is the crucial enzyme in the phenylpropanoid pathway [16]. As shown in Figure 1, the enzymatic activities of the typical defense response-related proteins increased significantly after the treatment of CurdOs proving that the CurdOs have definite elicitation effect on potato leaf cells.

Although the SA concentration and GLU activity (both at 4 h after elicitation) in leaf cells treated with CurdOs were experimentally higher than those treated with Lam, the differences of CTN and PAL activities on the CurdOs set were not significant from the Lam set. Consequently, further study is necessary to investigate the difference of mechanism among various elicitors.

A series of early- and late-defense responses were detected in potato foliar tissues (Fig. 2). ANOVA analysis shows that DP 5 (glucopentaose) exhibits the same elicitation activity as DP 6 and DP 7 but stronger than DP 4 (Fig. 4). Previous study performed in tobacco plants confirmed the elicitor activity of laminaripentaose [33], which supports our conclusion in this study.

### Linear glucopentaose and higher DP molecules activate an integrated response in potato leaf cells

The 2D-PAGE methodology was applied to determine the protein expression in potato foliar tissues treated by CurdOs (Fig. 5 and 6). A total of 10 proteins were identified by MALDI-TOF/TOF mass spectrometry (Table 1). Based on their basic functions [22], [30], [37], these proteins were classified into several categories, including metabolism, transcription, cell structure and disease/defense.

Three proteins directly involved in the disease-resistance reactions. The protein (No. 6605) was identical to an endochitinase belonging to the glycosyl hydrolases family 19 with a type 1 chitin binding domain (gi|1705805). The other two proteins were protease inhibitors which played positive roles on cell defense against pathogens [38]–[39]. The z-type serpin (No. 4405) was identical with the protein in *Hordeum vulgare* subsp. *vulgare* corresponding to clade P of the serpin superfamily which performed the inhibitory activity against cathepsin G [40]. The last one was identified as a putative Kunitz-type invertase inhibitor capable of inhibiting soluble tuber invertase of potato plant *in vitro* [41].

The metabolic-, transcriptional-, and cellular structural-related proteins were also up-regulated (Fig. 6). Leucine aminopeptidase (LAP, No. 2502) acts as an exopeptidase involving in protein turnover during cell growth [42]. LAPs participate in the defense response in tomato [43] and other plants [42] to breakdown exogenous proteins. L-Arginine is the precursor for the formation of many plant alkaloids [44], which is important for plant protection. As a crucial enzyme in the L-Arginine biosynthetic pathway [45], it is conceivable that the acetylornithine deacetylase (No. 2704) is up-regulated to cope with the elicitation of CurdOs.

Thioredoxin peroxidase (No. 1304) was identical to the protein in *Nicotiana tabacum* (gi|21912927) which conferred the role during elicitation in detoxification of hydrogen peroxide [46]–[47]. Similar activity has been found in *Capsicum annuum* during hypersensitive response to *Xanthomonas campestris* pv. *vesicatoria* [48]. The homeobox proteins and kinesins participate in the regulation of plant growth [49]–[51] and transportation of membranous organelles and protein complexes along with the microtubules within the cells [52]–[54], respectively. The function of neurochodrin-like protein (No. 501) is not clear at present in potato tissues whereas it leads to dendrite outgrowth in animal cells [55].

The biochemical and proteomic analyses indicate that the plant defense response is an integrated reaction including transduction of exotic elicitor signals and expression, transportation as well as relocation of defense-related proteins.

### Curdlan oligosaccharides activate the short-term defense responses

As shown in Figure 7, the lesion area of potato leaf decreased when the potato plants were treated with CurdOs 1 d before *P. infestans* infection. This result is agreement with the biochemical and proteomic analyses that the potato cells exhibited defense responses after elicitation of CurdOs. Conceivably, the defense reactions restrained the growth of *P. infestans* in the leaves.

However, the elicitation effect of CurdOs faded away with time. After 20 d of elicitation, both the GLU and PAL activities declined to the same level as the blank control. The difference between the CurdOs-treated sets and CurdOs-free set were not significant (Fig. 3). Additionally, the yield of potato plants treated with CurdOs at 5 d before infection was similar to the pathogen-infected control (*p* = 0.146) (Fig. 8), confirming the effectiveness of defense response in potato plants declined with time.

Our experimental results show the rapid elicitation effect of CurdOs but only for a short period of time on the induced defense response in potato plants. This conclusion is similar to the previous reports which used different plants as models [33], [56]–[58]. It appears that the potato plant gets accommodative to the stimulation after prolonged exposure to elicitors. Therefore, repeated uses of the elicitors are necessary method for protecting the potato plants from the infection of *P. infestans* at the present time.

## Supporting Information

Figure S1 **Quantitation of the spot intensity of proteins on the 2D-PAGE gel.** The color intensity of protein spot was used for quantification. The color intensity on the gel before elicitation was set as 1.0, CK. The color intensity after 12 h elicitation was normalized by CK. DP, degree of polymerization of curdlan oligosaccharide. All the values were the average of two replicates.
(TIF)
