# Supplementary figures and images for: Curdlan β-1,3-Glucooligosaccharides Induce the Defense Responses against Phytophthora infestans Infection of Potato (Solanum tuberosum L. cv. McCain G1) Leaf Cells
Source: PLoS One. 2014 May 9;9(5):e97197. doi: 10.1371/journal.pone.0097197 (PMC4016274; doi:10.1371/journal.pone.0097197)

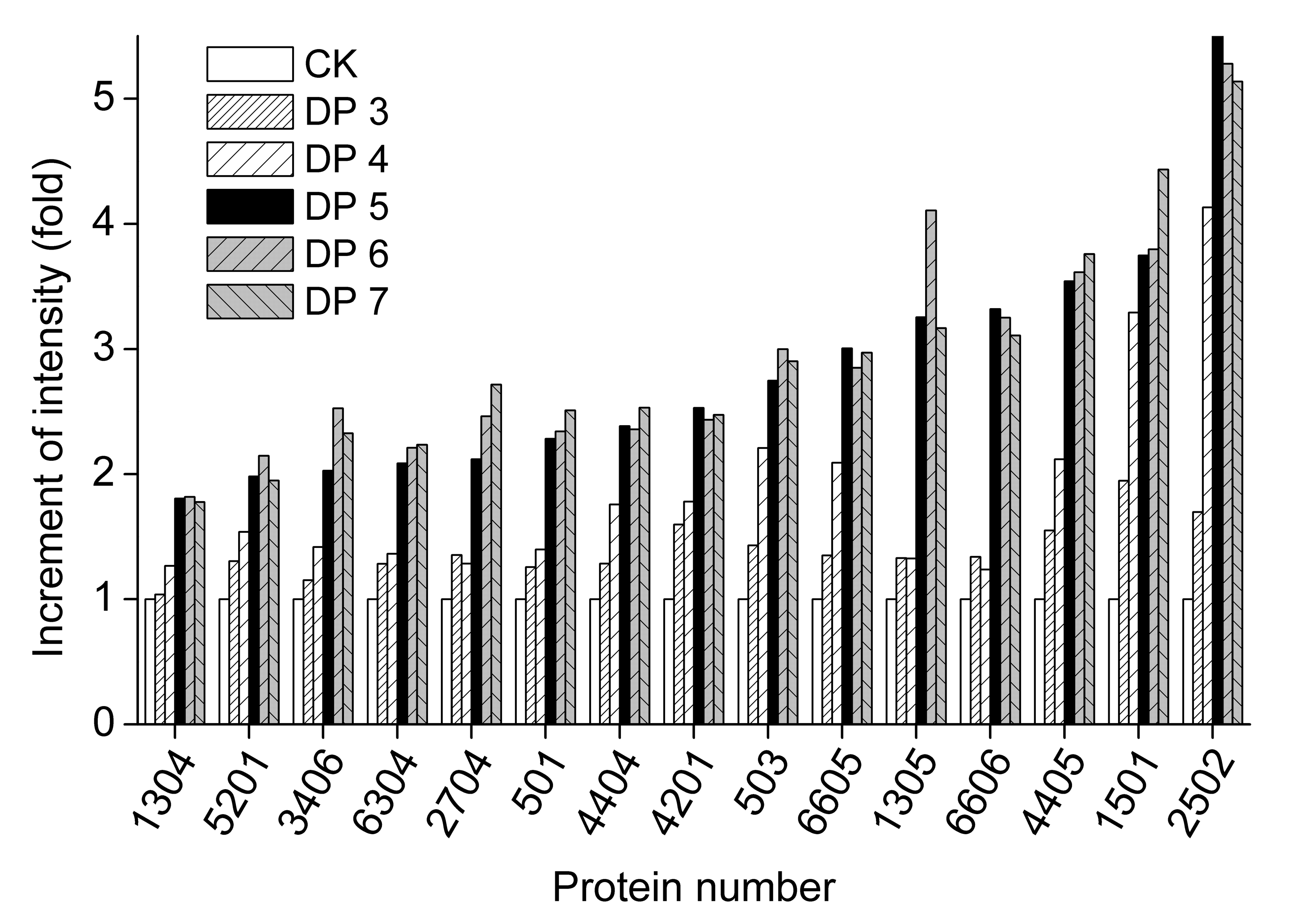

Supplement: Figure S1 — Quantitation of the spot intensity of proteins on the 2D-PAGE gel. The color intensity of protein spot was used for quantification. The color intensity on the gel before elicitation was set as 1.0, CK. The color intensity after 12 h elicitation was normalized by CK. DP, degree of polymerization of curdlan oligosaccharide. All the values were the average of two replicates. (TIF) [file pone.0097197.s001.tif]
